# A new simplified comorbidity score as a prognostic factor in non-small-cell lung cancer patients: description and comparison with the Charlson's index

**DOI:** 10.1038/sj.bjc.6602836

**Published:** 2005-10-18

**Authors:** B Colinet, W Jacot, D Bertrand, S Lacombe, M-C Bozonnat, J-P Daurès, J-L Pujol

**Affiliations:** 1 Thoracic Oncology Unit, Centre Hospitalier Universitaire de Montpellier, Hôpital Arnaud de Villeneuve, 34295 Montpellier Cedex 5, France; 2 Department of Statistics and Epidemiology, University Institute for Clinical Research, Hôpital Universitaire Arnaud de Villeneuve, France

**Keywords:** non-small-cell lung cancer, comorbidities, prognosis

## Abstract

Treatment of non-small-cell lung cancer (NSCLC) might take into account comorbidities as an important variable. The aim of this study was to generate a new simplified comorbidity score (SCS) and to determine whether or not it improves the possibility of predicting prognosis of NSCLC patients. A two-step methodology was used. Step 1: An SCS was developed and its prognostic value was compared with classical prognostic determinants in the outcome of 735 previously untreated NSCLC patients. Step 2: the SCS reliability as a prognostic determinant was tested in a different population of 136 prospectively accrued NSCLC patients with a formal comparison between SCS and the classical Charlson comorbidity index (CCI). Prognosis was analysed using both univariate and multivariate (Cox model) statistics. The SCS summarised the following variables: tobacco consumption, diabetes mellitus and renal insufficiency (respective weightings 7, 5 and 4), respiratory, neoplastic and cardiovascular comorbidities and alcoholism (weighting=1 for each item). In step 1, aside from classical variables such as age, stage of the disease and performance status, SCS was a statistically significant prognostic variable in univariate analyses. In the Cox model weight loss, stage grouping, performance status and SCS were independent determinants of a poor outcome. There was a trend towards statistical significance for age (*P*=0.08) and leucocytes count (*P*=0.06). In Step 2, both SCS and well-known prognostic variables were found as significant determinants in univariate analyses. There was a trend towards a negative prognostic effect for CCI. In multivariate analysis, stage grouping, performance status, histology, leucocytes, lymphocytes, lactate dehydrogenase, CYFRA 21-1 and SCS were independent determinants of a poor prognosis. CCI was removed from the Cox model. In conclusion, the SCS, constructed as an independent prognostic factor in a large NSCLC patient population, is validated in another prospective population and appears more informative than the CCI in predicting NSCLC patient outcome.

Guidelines for the treatment of non-small-cell lung cancer (NSCLC) have been proposed and, although not universally accepted, might contribute to the improvement of disease outcome. They are particularly useful for patients who are not eligible for clinical trials. The application of guidelines is a milestone in epidemiological observation and knowledge of the disease prognostic evolution. Daily practice, however, suggests that comorbidities in the therapeutic decision, although not conventionally defined, could be of paramount importance.

The awareness of prognostic determinants of NSCLC may be important in both clinical trials and routine practice (Komaki *et al*, 1993; Charloux *et al*, 1997; Paesmans *et al*, 1997; Merrill *et al*, 1999). For clinical trials, stratification of randomisation on known prognostic factors is an important part of procedure. In routine practice, therapeutic decision might be influenced by the state of prognostic variables (Komaki *et al*, 1993). Up to now, the most widely accepted prognostic determinants of NSCLC are disease stage and performance status (Brechot *et al*, 1996; Mountain, 1997). Several other features such as male gender, age older than 60 years, nonsquamous histologies have also been reported as negative prognostic factors (Williams *et al*, 1981; Charloux *et al*, 1997). In order to define NSCLC prognostic factors, simultaneous appraisal of eventual determinants could be carried out for a large patient population.

Routine treatment of patients with lung cancer requires taking into account smoking-related diseases, functional status and comorbidities. The term ‘comorbidity’ refers to noncancer-related physical and mental disorders that may also affect a patient outcome and treatment safety. Comorbidity should be distinguished from functional status, because the latter is a measure of a patient's ability to perform daily activities or other tasks (Firat *et al*, 2002a). Comorbidities may prevent the physician from delivering optimal therapy because of possible treatment-related side effects. Furthermore, for cancer in the elderly, comorbidities can have a major impact on survival (Extermann *et al*, 1998). As a result, patients who are eligible for conventional lung cancer studies generally meet ‘good general health criteria’ and represent a small segment of the NSCLC patient population routinely treated in cancer units. Moreover, several investigators in oncology consider comorbidities as a criterion for noneligibility. Restrictive eligibility criteria increase the certainty that any observed differences are attributable to the treatment and not to the confounding influence of comorbid diseases.

The prognostic significance of comorbidities, independent of performance status and tumour stage, has been extensively demonstrated in different types of cancer (Feinstein *et al*, 1977; Wells *et al*, 1984; Clemens *et al*, 1986; Miller *et al*, 1992; Waldman and Potter, 1992; Piccirillo *et al*, 1994; Satariano and Ragland, 1994; Piccirillo and Feinstein, 1996; Rochon *et al*, 1996; Pugliano *et al*, 1997; Singh *et al*, 1997; Extermann *et al*, 1998) including lung cancer (Feinstein and Wells, 1990; Firat *et al*, 2002a). Comorbidities may explain in part the variability of survival observed for example in stage I NSCLC, varying from 43 to 84%.

Alternatively, comorbid conditions have been evaluated using clinical scores in longitudinal studies. The most widely used clinical score is Charlson's. The Charlson comorbidity index (CCI), published by Charlson *et al* (1987) in 1987, was developed based on a longitudinal study of 559 patients admitted to a medical service during a 1-month period. Any disease or clinical condition resulting in a relative risk of death greater than 1.2 was included in the scale. A total of 19 conditions were found to influence significantly survival and were given a weighted score based on the relative mortality risk. The sum of the weighted scores of all of the comorbid conditions present in patients was then scaled to establish the CCI. The weighted index was tested for its ability to predict mortality in a cohort of women with histologically proven primary breast cancer. With each increased level of the comorbidity index, there was a stepwise increase in the cumulative mortality attributable to comorbid disease (Charlson *et al*, 1987).

We investigated the prognosis of a large NSCLC population followed-up over a substantial period of time, simultaneously assessing the aforementioned comorbidities together with classical prognostic determinants. This study aimed at determining whether or not the amalgamation of a new *simplified comorbidity score* (*SCS*) and the work up of NSCLC patients would improve the ability to predict prognosis and to compare this new score with the CCI. A two-step methodology was used in order to achieve this goal; firstly, a score elaboration step from a large database, used to build the SCS as a prognostic variable; secondly, a score validation step in which the SCS reliability was tested in an independent population.

## PATIENTS AND METHODS

### Step 1: score generation

#### Patients

In order to organise lung cancer care, we built in 1998 a health network of cancer institutions following common guidelines and willing to implement a prospective patient database (OncoLR). This health network was geographically limited to five French territorial divisions. One of the main end points was to describe accurately the characteristics of patients treated for NSCLC and to define their prognosis. In this multicentre prospective study, 735 consecutive patients were treated between January 1998 and May 2003 in the different institutions of the OncoLR health network.

All institutions were invited to input electronic case report forms into a comprehensive database owned by the oncoLR health network. Case reports extracted for this study were selected pending on the following criteria: histologically proven and previously untreated NSCLC. Consequently, patients suffering from small-cell lung cancer and patients admitted for adjuvant treatment (following surgery), second-line therapy or palliative care following anticancer treatment failure were not eligible. Histological subclassification was carried out according to the World Health Organization (WHO) classification (World Health Organization, 1982). Performance status was estimated according to the Eastern Cooperative Oncology Group (ECOG) (Zubrod *et al*, 1960) and the percentage of weight loss during the previous 4 months was recorded. Staging was carried out by exhaustive procedures according to the 4th edition of the *Union Internationale Contre le Cancer* (UICC) tumour node metastases (TNM) classification (Sobin *et al*, 1987), the American Thoracic Society map of regional pulmonary nodes (Tisi *et al*, 1982) and the new Mountain stage grouping (Mountain, 1997). The following investigations were carried out: a clinical examination, a standard chest roentgenography, a computed tomographic (CT) scan of chest and upper abdomen, fibreoptic bronchoscopy, liver sonography and bone scanning. A mediastinoscopy was used to establish nodal status in NSCLC patients without evident haematogeneous metastatic disease and evidence of mediastinal lymph node enlargement on chest CT-scan. A brain CT-scan was performed routinely. The upper limits of normal values were as follows: leucocytes: 10 000 *μ*l^−1^; platelets count: 400 000 *μ*l^−1^.

This part of the study aimed at generating a new simplified comorbidity score using specific comorbidities definitions. Both academic institutions and community hospitals were involved in this pragmatic part of the study, and the complex CCI that requires the assessment of a broad number of comorbidities was not included in step 1.

#### Comorbidity item definition

Cardiovascular comorbidity was defined as the presence of one or more of the following: congestive heart failure, ischaemic cardiopathy with or without myocardial infarction, severe valvular cardiopathy, arrhythmia requiring chronic treatment, history of cerebrovascular disease, hypertension and/or peripheral vascular disease. Respiratory comorbidity was defined as the presence of one or more of the following: history of tuberculosis, history of pleural effusion or pneumonia, asthma, pulmonary embolism, chronic pulmonary insufficiency as defined by a chronic hypoxemia less than 60 mmHg and/or chronic obstructive pulmonary disease (COPD) inducing a FEV_1_ less than 1.5 l. Neoplasic comorbidities were defined as a previous personal history of cancer, excluding basal cell carcinoma of the skin and *in situ* carcinoma of the cervix. Renal insufficiency was defined as a creatinine clearance lower than 60 ml min^−1^ (calculated according to the Cockroft formula). Diabetes mellitus was defined as diabetes treated with either oral hypoglycemics or insulin. Alcoholism was defined as a daily consumption of more than 80 g of alcohol for men and 40 g for women. Tobacco consumption was defined as a lifelong consumption of an equivalent of at least 100 cigarettes.

#### Item weighting and elaboration of the SCS

The following method was adopted in order to build the SCS: comorbidities were tested as single variables in independent univariate analysis. The relative risk of death was taken into account in order to select and organise the variables to be tested in an initial multiparametric survival analysis. Each variable was tested in the multivariate analysis and the *β* coefficients were determined with regards to the adjusted risk. As a result of this analysis, the major comorbidities were tobacco consumption, diabetes mellitus and renal insufficiency as described above. These three variables were affected by the highest weightings (7, 5 and 4, respectively). Other features included in the SCS were respiratory, neoplasic and cardiovascular comorbidities and alcoholism. Those well-known limiting factors in lung cancer management were affected by a weighting of 1 (Table 1).

### Step 2: score validation

The SCS reliability as a prognostic determinant was tested in a different population of 136 NSCLC patients prospectively accrued in our two university departments from September 2003 to June 2004. Eligibility criteria and pretherapeutic work-up were conducted similarly to the step 1 patient population. Additional variables tested were: haemoglobin level, blood lymphocytes count, serum fibrinogen, sodium, calcium, proteins, albumin, alkaline phosphatases, lactate dehydrogenase (LDH), Cyfra 21-1 and neuron-specific enolase (NSE) levels. Finally, the CCI was evaluated previous to any treatment.

### Treatment

Treatment was conducted similarly for the two populations. A medical panel composed of thoracic surgeons, chest physicians, radiologists, radiotherapists and medical oncologists discussed the case of each patient in order to design a treatment programme to be submitted for patient's approval. Particular attention was paid to the agreement between each individual proposal and the oncoLR guidelines (http://poumon.oncolr.org/public/thesaurusPoumon.asp).

NSCLC patients with stage I or II disease underwent surgery in an attempt at complete resection. Patients suffering from pathologically demonstrated N2 disease received cisplatin-based neoadjuvant chemotherapy followed by surgery whenever possible. Other patients with performance status ⩽2 and distant metastases (stage IV) or gross mediastinal involvement (stage IIIb and unresectable stage IIIa) were treated when clinically possible by a cisplatin-based chemotherapy. Radiotherapy was applied in locally advanced stages according to a concomitant schedule (Furuse *et al*, 1995). Best supportive care, including palliative radiation-therapy when needed, was proposed to patients with advanced stage and poor performance status. Treatment was decided upon according to clinical and routine biological findings and without knowledge of the SCS, although some of the comorbidities were obviously taken into account in therapeutic choice (eg poor respiratory function and surgical contraindication). Hence, treatment was not considered as a prognostic variable in this study.

### Statistics

*κ* coefficient of reliability (Snedecor and Cochran, 1956) and McNemar test of symmetry (Armitage, 1971) were used to test the concordance of the two comorbidity scales that is, CCI and SCS. A *P*-value <0.05 was considered as significant.

Survival was defined as the time from database registration to the date of death whatever the cause. For the step 1 population, survival data were updated on 1 September 2003. At end point, 14 patients were lost to follow-up (1.9%). Median follow-up was 25.9 months (range 3.3–67.4 months) and 530 events were recorded. For the step 2 population, survival data were updated on 14 January 2005 and none of the patients were lost to follow-up. Median follow-up was 10.3 months (range 6.7–16.5 months) and 50 events were recorded.

Coding methods for the different variables depended on their nature. Some of the variables were extensively described in the literature, therefore the threshold was defined using previous publications. Performance status was analysed according to two classical modalities: PS 0–1 and PS greater or equal to 2 (Zubrod *et al*, 1960). The effect of nodal status on prognosis was tested according to the presence or the absence of mediastinal lymph node involvement. The same coding regarding tumour status has been adopted according to the new Mountain's stage grouping (stages I–IIIa *vs* stages IIIb–IV) (Mountain, 1997). Owing to the fact that the OncoLR guidelines are based on stage grouping according to the Mountain's system rather than the detailed TNM, we considered Montain's stage grouping as the staging variable in the Cox model. TNM was not introduced in order to avoid statistical redundancy. For the biological variables, previously published thresholds were used particularly for Cyfra 21-1 (Pujol *et al*, 1993, 2001) and NSE serum levels (Jorgensen *et al*, 1996; Pujol *et al*, 2001).

Probability of survival was estimated by the Kaplan and Meier (1958) method. Single variable survival analyses was assessed by means of the Wilcoxon and log-rank tests and multivariate regression was assessed with Cox's model (Cox, 1972; Andersen, 1991). The classical forward selection of variable procedure was used. The variables to be tested in the Cox model were selected using the results of univariate analyses, that is, variables reaching at least a *P* level less than 15%. This model was written after a Boolean coding of the significant variables: categorical variables (such as performance status) were transformed into binary variables (0: negative or 1: positive). The number of levels of a Boolean variable needed to describe a predictive factor is one less than the categories of that factor inasmuch as its baseline level is defined by setting the value of each of the Boolean variables at zero. The significance of the effect of a given factor was assessed by determining whether or not the coefficient assigned to one or more of its categories was sufficiently different from zero. The proportional hazard assumption for each of the selected variables retained in the final model was initially checked by plotting the log cumulative baseline hazard ratio. A *P* level of less than 0.05 was considered significant. All tests were two-sided. Survival was analysed using the SAS software package.

In the step 1 population, the above-mentioned procedure identified eight variables as putative prognostic determinants to be tested in the Cox regression hazard model whereas in the step 2 population the number of variables was 17. Therefore, the main population complied with the current recommendation (Harrell *et al*, 1985) insofar as the number of variables represented less than 10% of the total of observed events (530 deaths).

## RESULTS

### Step 1: score generation

Patient's demography and disease characteristics are summarised in Table 2. Most of the main characteristics of NSCLC were retrieved particularly a median age of 62.5 years. The median survival of the whole population was 12.7 months (95% CI, 11.2–14). The 1- and 2-year survival rates were 51% (95% CI, 48–55%) and 29% (95% CI, 26–33%), respectively.

#### Univariate analysis

The univariate analysis (Table 3) showed that patients affected by one of the following characteristics proved to have a shorter survival in comparison with the opposite status of each variable: male gender, age ⩾70 years, performance status ⩾2, tumour status ⩾3, nodal status ⩾2, metastatic status (M1), stage grouping IIIb or IV, weight loss ⩾5%, blood leucocytes count >10 000 *μ*l^−1^, blood platelets count >400 000 *μ*l^−1^, current or former smoker, renal insufficiency and SCS greater than 9.

#### Multivariate analysis

The following variables were independent determinants of a poor outcome (Table 4): weight loss ⩾5%, hazard ratio (95% confidence interval): 1.39 [1.12–1.73], stage grouping: 2.46 [1.90–3.18], performance status: 1.33 [0.99–1.76] and SCS: 1.36 [1.09–1.69]. Two additional variables did not formally reach statistical significance level: age ⩾70 years: 1.23 [0.98–1.54] (*P*=0.08) and leucocytes: 1.23 [0.99–1.52] (*P*=0.06).

As a control, the SCS was tested as a continuous variable and the Cox model was run again with the same variables. In this model, SCS was retained and affected by a 1.05 hazard ratio [1.02–1.08], *P*=0.002. Other variables were similar with nearly identical hazard ratios.

### Step 2: score validation

Patient population and disease characteristics are summarised in Table 5 together with the main univariate survival results. Patient population in steps 1 and 2 of this study shared similar characteristic distributions in terms of main prognostic variables. the 1-year survival rate was 59% and median survival was not reached at the time of study analysis.

#### Concordance of two comorbidiy scales

A statistical concordance was observed between CCI and SCS (*κ* coefficient of reliability=0.288; *P*<0.00001; Table 5). There was a statistically significant asymmetry (McNemar test of symmetry; *P*=0.0008).

#### Univariate analysis

**Figure 1:**
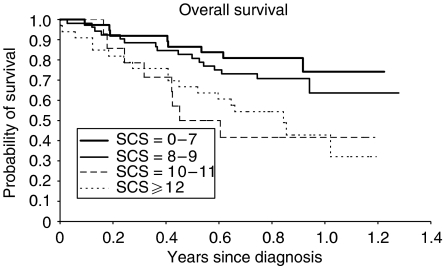
Step 2 patients survival according to SCS (log rank test; *P*<0.01).

**Figure 2:**
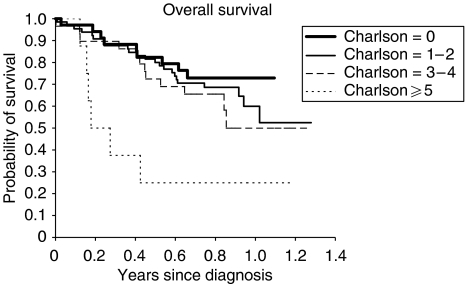
Step 2 patients survival according to CCI (log rank test; *P*=0.06).

The univariate analysis demonstrated a significant shorter survival for patients presenting one of the following characteristics in comparison with patients presenting the opposite status (Table 6): age >70 years, performance status ⩾2, stage grouping IIIb–IV, weight loss ⩾5%, leucocytes >10 000 *μ*l^−1^, nonadenocarcinomatous histology, alkaline phosphatase ⩾104 U l^−1^, high CYFRA 21-1 serum level, lymphocytes <1000 *μ*l^−1^, protein ⩽64 g l^−1^, albumin <35 g l^−1^, LDH⩾450 U l^−1^, NSE >12.5 ng ml^−1^, serum calcium >2.6 mmol l^−1^, anaemia, high fibrinogen level and SCS >9. In addition, there was a trend towards a significant negative prognostic effect for a CCI⩾3, platelets >400 000 *μ*l^−1^ and a serum sodium ⩽135 mEq l^−1^. Survival according to CCI and SCS values are shown in Figures 1 and 2, respectively.

#### Multivariate analysis

In the multivariate analysis, the following variables were independent determinants of a poor outcome: stage grouping, 9.03 [3.04–26.76], performance status, 2.92 [1.43–5.98], histology, 2.58 [1.21–5.48], leucocytes, 4.74 [2.30–9.76], lymphocytes, 2.93 [1.19–7.22], LDH, 3.48 [1.66–7.33], CYFRA 21-1, 3.77 [1.80–7.89], SCS, 2.66 [1.33–5.30]. However, CCI was removed from the Cox model. It is worth noting that SCS, as a new prognostic determinant, appeared as efficient as stage grouping in defining outcome and seems to replace the CCI most likely as a result of redundancy of the prognostic information.

## DISCUSSION

In the score generation part of this study (step 1), we evaluated the prognosis of a large NSCLC population accrued during a 5-year period, simultaneously assessing different aforementioned comorbidities together with classical prognostic determinants. The threshold value for this new score has been chosen taking into account a clear cutoff effect within and beyond the value 9 according to univariate analysis. The 1- and 2-year survival rates were 51 and 29%, respectively, with a median survival of the whole population of 12.7 months, which is consistent with NSCLC population survival curves. In the multivariate analysis, several characteristics were identified as independent poor prognosis variables: poor performance status, weight loss equal to or greater than 5%, stage equal to or greater than IIIb and SCS greater than 9. The first three are well-identified NSCLC features strongly indicating prognosis (Stanley, 1980; Paesmans *et al*, 1995). The main goal of the step 1 part of this study was to generate a simple comorbidity index, which may add new information to the prognostic patient equation. In the score validation part of this study (step 2), the SCS was tested in two ways: (i) by evaluating this new score together with a wide panel of prognostic variables including biological ones that were not exhaustively assessed in step 1, and (ii) by comparing SCS and CCI, the latter having been extensively applied to different malignant diseases including lung cancer (Firat *et al*, 2002a, 2002b; Pergolizzi *et al*, 2002; Birim *et al*, 2003a, 2003b; Ludbrook *et al*, 2003; Tammemagi *et al*, 2003). Both criteria were reached demonstrating the validity of the variables as a prognostic determinant.

The OncoLR heath network is devoted to cancer therapy and accrued all patients affected by lung cancer without other eligibility restriction than those defined in the Pateints and Method section, that is, histologically proven and previously untreated NSCLC. Physicians participating in the OncoLR heath network are mainly oncologists and chest physicians involved in cancer care. Therefore, there is a possibility that elderly patients with very poor presentation were refereed to other institutions such as palliative care units. Nevertheless, we consider that our population is more representative of overall lung cancer population than are for example populations included in clinical trials.

Comorbid conditions are frequent in NSCLC patient populations, considering the mean age and the high frequency of smokers of this population. Comorbidities are considered as an important prognostic factor in patients with different types of cancer, including lung cancer (Feinstein *et al*, 1977; Wells *et al*, 1984; Clemens *et al*, 1986; Feinstein and Wells, 1990; Miller *et al*, 1992; Waldman and Potter, 1992; Piccirillo *et al*, 1994; Satariano and Ragland, 1994; Piccirillo and Feinstein, 1996; Rochon *et al*, 1996; Pugliano *et al*, 1997; Singh *et al*, 1997; Extermann *et al*, 1998; Firat *et al*, 2002a). Comorbidities may impair survival by themselves or by affecting the therapeutic options. For example, the 5-year survival rate for the rectum (Feinstein *et al*, 1975), larynx (Feinstein *et al*, 1977), endometrial (Wells *et al*, 1984) or prostate (Clemens *et al*, 1986) carcinomas differs when a prognostic comorbidity is present (11, 15, 27, and 16%, respectively) or absent (32, 54, 78 and 60%). In our study, a high frequency of comorbidities has been found especially for respiratory and cardiovascular diseases, closely associated with smoking status. These results are consistent with other unselected series of lung cancer. For example, in a multicentre study of 2992 patients affected by an operable NSCLC, 73% presented with one or several of the tested comorbidities and 50% presented with an associated COPD (Lopez-Encuentra, 2002). Other experiences report comorbidity frequencies of up to 61% on 442 newly diagnosed cases of lung cancer (Schrijvers *et al*, 1997).

Age is generally considered as a negative prognostic factor, whatever the treatment modality (Stanley, 1980; Williams *et al*, 1981; Paesmans *et al*, 1995). However, there is no consensus regarding the threshold that might be used in order to define an elderly population. Ages varying from 60 to 70 years have been reported. In addition, there is a lack of epidemiological data concerning prognostic factors in the elderly NSCLC patient populations. In our study, age >70 years was associated with a worse outcome, reaching statistical significance in an univariate analysis. It is worth noting that this variable did not reach conventional statistical significance in the multivariate analysis suggesting that there is redundancy of the herein SCS and age.

Comorbid conditions have been evaluated using several clinical scores from longitudinal studies. The most widely used clinical score is the CCI, developed by Charlson *et al* (1987). This index has been validated in a cohort of breast cancer patients (Charlson *et al*, 1987), giving a method of measuring the prognostic impact of comorbid disease. Since this initial publication, the CCI has been validated and used in several cancer studies (head and neck (Singh *et al*, 1997), stomach (Lubke *et al*, 2003), bladder (Miller *et al*, 2003), kidney (Gettman *et al*, 2003), prostate (Froehner *et al*, 2003)) including lung cancer (Firat *et al*, 2002a; Birim *et al*, 2003a) and cancer in the elderly (Extermann *et al*, 1998). In our study, we developed and validated a new SCS. This score considers tobacco consumption together with clinical comorbid conditions (diabetes mellitus, renal insufficiency, respiratory, neoplasic and cardiovascular comorbidities) and alcoholism. An SCS greater than 9 was found to be an independent prognostic factor of poor outcome. This score has been developed for a large, unselected NSCLC population. An advantage of SCS over standard comorbidity scores including CCI is the reduced number of items taken into account allowing score calculation in only a few minutes. As not all comorbidities assessed in the CCI were checked in the SCS, we considered that there was a need for direct comparison of the SCS and the CCI in the same population. This evaluation was formally carried out in the score evaluation part of the study. In this second population, the SCS was validated as an independent prognostic determinant and appears more informative than the CCI in predicting patient outcome in the setting of NSCLC patients.

In conclusion, the SCS, constructed as an independent prognostic factor in a large NSCLC patient population, is validated in another prospective population and appears more informative than the CCI in predicting patient outcome. The amalgamation of this new prognostic score into the set of classical prognostic determinants might be useful when prognostic studies are evaluated. SCS could also be considered as a part of the pretherapeutic work-up of clinical trials. The Cox model result of the second step is a clue in favour of the independent negative prognostic effect of poor comorbidity score. This result deserves further studies designed to determine whether or not this finding has clinical impact in treatment algorithms.

## Figures and Tables

**Table 1:** The simplified comorbidity score and weighting of comorbidities

**Comorbidity**	**Weighting**
Tobacco consumption	7
Diabetes mellitus	5
Renal insufficiency	4
Respiratory comorbidity	1
Cardiovascular comorbidity	1
Neoplastic comorbidity	1
Alcoholism	1

**Table 2:** Step 1 patients' demography and disease characteristics

**Variables**	**No. of patients (%)**
Total	735
*Age (years)*	
Median±s.d.	62.5±11.2
	
*Gender*	
Male	589 (80)
Female	146 (20)
	
*ECOG performance status*	
<2	606 (82)
⩾2	124 (17)
Missing data	5 (1)
	
*T-stage*	
1–2	245 (33)
3–4	466 (63)
Missing data	24 (4)
	
*N-stage*	
0–1	270 (37)
2–3	438 (60)
Missing data	27 (3)
	
*M-stage*	
0	395 (54)
1	340 (46)
	
*Stage grouping (Mountain)*	
Ia	9 (2)
Ib	35 (5)
IIa	1 (1)
IIb	48 (6)
IIIa	112 (14)
IIIb	181 (24)
IV	340 (46)
Missing data	9 (2)
	
*Histology*	
Squamous cell carcinoma	273 (37)
Adenocarcinoma	345 (47)
Large cell carcinoma	105 (14)
Unspecified NSCLC	12 (2)
	
*Weight loss* (%)	
<5%	392 (53)
⩾5%	220 (30)
Unknown	123 (17)
	
*Blood leuccyte count*	
⩽10 000 *μ*l^−1^	435 (60)
>10 000 *μ*l^−1^	233 (31)
Unknown	67 (9)
	
*Blood platelets count*	
⩽400 000 *μ*l^−1^	541 (75)
>400 000 *μ*l^−1^	130 (17)
Unknown	64 (8)
	
*Metastases*	
Adrenal gland	54 (7)
Bone	129 (18)
Liver	54 (7)
Lung	140 (19)
Brain	71 (10)
Other sites	36 (5)
	
*Smoking status*	
Current smoker	384 (52)
Former smoker	255 (36)
Non smoker	73 (9)
Missing data	23 (3)
	
*Comorbidities*	
Cardiovascular	265 (36)
Respiratory	320 (44)
Neoplasic	86 (12)
Diabetes mellitus	67 (9)
Alcoholism	113 (15)

**Table 3:** Step 1, univariate analysis

**Variable**	**Median survival (months) [95% CI]**	***P* (log-rank)**
*Age (years)*		
⩽70	14 [12.4–16]	<0.01
>70	9.6 [8.8–12]	
		
*Gender*		
Female	16.2 [13.9–21.6]	0.03
Male	11.7 [10.3–13.3]	
		
*ECOG performance status*		
<2	14.1 [12.7–15.7]	<0.01
⩾2	6.7 [4.3–8.1]	
		
*T-stage*		
1–2	18.5 [14.8–21.8]	<0.01
3–4	10.4 [9.6–12.3]	
		
*N-stage*		
0–1	16.3 [14–20]	<0.01
2–3	10.6 [9.6–12.3]	
		
*M-stage*		
0	18.2 [15.7–21.6]	<0.01
1	8.8 [7.8–10]	
		
*Stage grouping*		
I–IIIa	24.7 [20.3–27.5]	<0.01
IIIb–IV	10.2 [9.4–11.5]	
		
*Histology*		
Adenocarcinoma	14 [11.3–16.2]	0.31
Nonadenocarcinomatous	11.8 [10.3–13.5]	
		
*Weight loss*		
<5%	16.5 [13.9–18.5]	<0.01
⩾5%	8.7 [7.2–10.4]	
		
*Blood leucocyte count*		
⩽10 × 10^9^ l^−1^	14.7 [12.8–17]	<0.01
>10 × 10^9^ l^−1^	10.4 [9.5–13.2]	
		
*Blood platelets count*		
⩽400 × 10^9^ l^−1^	13.5 [12.1–15]	0.03
>400 × 10^9^ l^−1^	9.9 [8.7–14]	
		
*Smoking status*		
Smoker	12.3 [10.9–13.8]	0.04
Nonsmoker	16.2 [13.3–24.3]	
		
*Cardiovascular comorbidities*		
Yes	13.2 [11.4–14.8]	0.62
No	11.7 [9.8–14.4]	
		
*Respiratory comorbidities*		
Yes	13 [10.8–14.9]	0.16
No	12.4 [10.5–14.2]	
		
*Neoplasic comorbidities*		
Yes	12.9 [11.3–14.1]	0.96
No	12 [9.7–18.2]	
		
*Diabetes mellitus*		
Yes	12.9 [11.3–14.1]	0.08
No	10.3 [7.4–15]	
		
*Renal insufficiency*		
Yes	13.8 [12.4–15.7]	0.02
No	10.4 [9–14.2]	
		
*Alcoholism*		
Yes	13.3 [11.3–14.6]	0.07
No	10.4 [8.4–13.8]	
		
*Simplified comorbidity score*		
⩽9	14.7 [13–16.9]	<0.01
>9	9.9 [8.6–13.2]	

**Table 4:** Step 1, estimated hazard ratios for significant variables

**Variables**	**Hazard ratio**	**95% CI**	***P*-value**
Mountain's stage IIIb–IV	2.46	[1.90–3.18]	<0.001
Simplified comorbidity score	1.36	[1.09–1.69]	0.006
Weight loss more than 5%	1.39	[1.12–1.73]	0.003
Poor Performance Status	1.33	[0.99–1.76]	0.050
Age >70 years	1.23	[0.98–1.54]	0.080
Leucocytes >10 000 *μ*l^−1^	1.23	[0.99–1.52]	0.060

**Table 5:** Step 2, Charlson comorbidity index (CCI) *vs* simplified comorbidity score (SCS): classification of comorbididy conditions by both scales (*κ* coefficient of reliability=0.288; *P*<0.00001; McNemar test of symmetry; *P*=0.0008)

	**SCS**				
**CCI**	**0–7**	**8–9**	**10–11**	**⩾12**	**Total**
0	24	7	2	1	34
1–2	11	35	3	16	65
3–4	2	9	5	13	29
⩾5	0	1	4	3	8
					
Total	37	52	14	33	136

**Table 6:** Step 2 patients' demography, disease characteristics and univariate analysis

**Variable**	**Number**	**Median survival (months) [95% CI]**	***P* (log-rank)**
Total	136		
*Age (years, median±s.d.)*			
62.5±11.2			
			
*Age (years)*			
⩽70	92 (67.6)	NR [12.3–NR]	0.05
>70	44 (32.4)	11.3 [6.0–NR]	
			
*Gender*			
Female	29 (21.3)	NR [NR–NR]	0.44
Male	107 (78.7)	NR [11–NR]	
			
*ECOG performance status*			
<2	105 (77.2)	NR [12.3–NR]	<0.01
⩾2	31 (22.8)	6.3 [4.9–NR]	
			
*Stage grouping*			
I–IIIa	52 (38.5)	NR [NR–NR]	<0.01
IIIb–IV	84 (61.5)	10.1 [6.4–NR]	
			
*Histology*			
Adenocarcinoma	58 (42.6)	NR [NR–NR]	<0.01
Nonadenocarcinomatous	78 (57.4)	11.3 [7.8–NR]	
			
*Weight loss*			
<5%	77 (56.6)	NR [NR–NR]	0.02
⩾5%	59 (43.4)	11.3 [7.3–NR]	
			
*Blood leucocyte count*			
⩽10.10^9^ l^−1^	86 (63.2)	NR [NR–NR]	<0.01
>10.10^9^ l^−1^	50 (37.8)	7.9 [4.9–NR]	
			
*Haemoglobin level*			
⩽11 g dl^−1^	20 (14.7)	6.4 [2.9–10.3]	<0.01
>11 dl^−1^	116 (85.3)	NR [12.3–NR]	
			
*Blood lymphocyte count*			
⩾10^9^ l^−1^	118 (86.8)	NR [NR–NR]	<0.01
<10^9^ l^−1^	13 (9.6)	4.4 [2.0–12.3]	
			
*Blood platelets count*			
⩽400.10^9^ l^−1^	109 (80.1)	NR [12.3–NR]	0.08
>400.10^9^ l^−1^	27 (19.9)	10.3 [6.0–NR]	
			
*CCI*			
<3	99 (72.8)	NR [12.3–NR]	0.06
⩾3	37 (27.2)	10.3 [5.4–NR]	
			
*SCS*			
⩽9	89 (65.4)	NR [NR–NR]	<0.01
>9	47 (34.6)	10.1 [5.4–NR]	
			
*Serum fibrinogen level*			
Normal	41 (30.1)	NR [NR–NR]	<0.01
Increased	86 (69.9)	11.3 [7.8–NR]	
			
*Serum calcium level*			
Normal	130 (97.1)	NR [12.3–NR]	0.02
Increased	4 (2.9)	5.2 [0.1–NR]	
			
*Serum sodium level*			
Normal	122 (89.7)	NR [12.3–NR]	0.09
Lowered	14 (10.3)	7.2 [2.1–NR]	
			
*Alkaline phosphatases*			
Normal	97 (71.3)	NR [NR–NR]	<0.01
Increased	36 (26.5)	7.4 [4.9–NR]	
			
*LDH*			
Normal	104 (76.5)	NR [NR–NR]	<0.01
Increased	28 (20.6)	5.9 [4.4–NR]	
			
*Serum protein level*			
⩽64 g l^−1^	10 (7.4)	NR [NR–NR]	<0.01
>64 g l^−1^	125 (91.9)	4.2 [1.1–12.3]	
			
*Serum albumin level*			
<35 g l^−1^	18 (13.2)	5.9 [2.7–NR]	<0.01
⩾35 g l^−1^	115 (84.6)	NR [12.3–NR]	
			
*Serum Cyfra 21–1 level*			
<3.6 ng ml^−1^	73 (53.7)	NR [NR–NR]	<0.01
⩾3.6 ng ml^−1^	58 (42.6)	7.4 [6.3–NR]	
			
*Serum NSE level*			
⩽12.5 ng ml^−1^	83 (61.0)	NR [12.3–NR]	0.01
>12.5 ng ml^−1^	38 (27.9)	7.4 [5.4–NR]	

CCI: Charlson comorbidity index; ECOG: Eastern Cooperative Oncology Group; LDH: lactate dehydrogenase; NR: not reached; NSE: neuron-specific enolase; SCS: simplified comorbidity score; s.d.: standard deviation.
